# Impact of Erythritol Air‐Polishing on Titanium Implant Surface Properties and Bacterial Colonization: An In Vitro Study

**DOI:** 10.1002/cre2.70289

**Published:** 2026-01-14

**Authors:** Stefano Sivolella, Giulia Brunello, Enrico Lotta, Michele Stocchero, Roberto Meneghello, Paola Brun

**Affiliations:** ^1^ Department of Neuroscience, Dentistry Section University of Padova Padova Italy; ^2^ Department of Oral Surgery University Hospital Düsseldorf Düsseldorf Germany; ^3^ Department of Orthodontics and Dentofacial Orthopedics Charité ‐ Universitätsmedizin Berlin, corporate member of Freie Universität Berlin and Humboldt‐Universität zu Berlin Berlin Germany; ^4^ Department of Oral & Maxillofacial Surgery and Oral Medicine, Faculty of Odontology Malmö University Malmö Sweden; ^5^ Department of Management and Engineering University of Padova Padova Italy; ^6^ Department of Molecular Medicine University of Padova Padova Italy

**Keywords:** antimicrobial, bacterial adhesion, dental implant, erythritol, powder

## Abstract

**Objectives:**

This study aimed to investigate the effect of erythritol air‐polishing on implant surface topography and bacterial colonization, and to determine the antimicrobial activity of erythritol powder.

**Materials and Methods:**

Titanium implants, with machined/acid‐etched hybrid design, were divided into three groups: erythritol air‐polishing for 1 min (E1), 5 min (E5), and untreated control. Surface analysis was performed using a stylus profilometer and scanning electron microscope (SEM). To test the ability to prevent biofilm formation, four bacteria strains (*Staphylococcus aureus*, *Klebsiella pneumoniae*, *Streptococcus mutans*, *Streptococcus sanguinis*) were separately cultured on five implants per group and colony counting was performed. The intrinsic erythritol antibacterial activity was investigated by means of minimum inhibitory concentration against the same strains.

**Results:**

At SEM analysis implant surfaces appeared unaltered by air‐polishing and presented increasing amount of residues depending on the treatment duration. Machined surfaces exhibited no significant differences in roughness parameters between the groups. On acid‐etched surfaces, E5 presented significantly lower Ra (vs. E1 and control) and Rz (vs. control). The count of colonies was significantly lower for all bacterial strains on treated implants as compared to control, with E1 and E5 being equally capable to reduce by 1.5 log bacteria growth. Erythritol antimicrobial activity against all tested bacterial strains was confirmed.

**Conclusions:**

The proposed erythritol air‐polishing protocols did not alter implant surfaces and the antimicrobial properties of erythritol are conserved by the titanium implant surfaces.

**Clinical Relevance:**

Erythritol air‐polishing could be repeatedly used in supportive peri‐implant care programmes.

## 1 Introduction

Biofilm is a major contributor to peri‐implant diseases, as it contains bacteria that trigger inflammation in the surrounding tissues (Berglundh et al. 2018). Therefore, its removal plays a pivotal role in the management of peri‐implant diseases. A wide range of decontamination techniques have been developed and employed to effectively treat implant surfaces and disrupt bacterial biofilm, aiming to restore implant health and prevent disease progression, possibly without detrimental effect on implant surface. These include the use of manual instruments, brushes, air‐polishing, laser, sonic and ultrasonic devices (Brunello et al. 2025; Herrera et al. 2023; Monje et al. 2022; Sivolella et al. 2021). However, alterations provoked by these cleaning procedures in terms of roughness and hydrophilic properties may further promote bacterial recolonization, hindering the healing process and favoring the recurrence of the pathology (Delucchi et al. 2025).

Although there is insufficient clinical evidence to suggest that an implant decontamination technique is better than others, in vitro studies have shown that abrasive powders have a greater or equal decontamination potential than other methods, with the advantage of producing minor modifications of the implant surfaces compared to curettes or ultrasonic tips (Ichioka et al. 2022, 2023; Matsubara et al. 2020; Sahrmann et al. 2021; Toma et al. 2018).

Air‐polishing devices emit a jet of water, pressurized air and low‐abrasive microparticles able to remove the biofilm from teeth, implant surfaces and prosthetic components (Delucchi et al. 2025). Beside sodium bicarbonate, different powders characterized by lower grain size and abrasiveness are currently available, including glycine, erythritol, and trehalose (Delucchi et al. 2025; Kruse et al. 2022; Liu et al. 2024; Moharrami et al. 2019).

Air‐polishing with erythritol powder is gaining attention as an effective method for dental implant surface disinfection in supportive and preventive professional hygienic treatment (Delucchi et al. 2025; Liu et al. 2024). Erythritol is a biocompatible and non‐toxic substance, with a finer granulometry than glycine, that is 14 µm and 25 µm, respectively (Delucchi et al. 2025). It belongs to the group of polyols, such as xylitol, menthol, and sorbitol, and is present in small quantities in many fruits, including melons, peaches, grapes, and pears (Mazi and Stanhope 2023). Erythritol air‐polishing removes the biofilm and inhibits its formation, even though the mechanism behind the antibiofilm action of erythritol has not yet been clarified. Hypotheses have been postulated to explain this phenomenon, including the ability of erythritol to inhibit polysaccharide‐adhesion mediated by Streptococci or to suppress bacterial growth by depletion of DNA and RNA (Hashino et al. 2013; Loimaranta et al. 2020).

A commercially available formulation containing chlorhexidine (CHX) has been found to inhibit multispecies biofilm regrowth on implant surfaces as compared with mechanical removal by saline and gauze (Amate‐Fernández et al. 2021). The antibiofilm activity of this product has also been confirmed against clinical strains of *Staphylococcus aureus*, *P. aeruginosa*, *B. fragilis,* and *C. albicans* isolated from peri‐implantitis lesions (Drago et al. 2017). To what extent this antibiofilm properties can be attributed to the addition of 0.3% CHX is not known. However, this small amount of CHX is added by the manufacturer as a preservative and not for therapeutic purposes (Drago et al. 2017).

In accordance with the European Federation of Periodontology S3 level clinical practice guideline (Herrera et al. 2023), air‐polishing devices with glycine powder or erythritol alone or in combination can be used for professional mechanical plaque removal in the maintenance phase in patients treated for peri‐implantitis in order to diminish the risk of recurrence. Furthermore, the use of air‐polishing alone may be considered as non‐surgical mechanical therapy in patients with mucositis (Herrera et al. 2023). Whereas air‐polishing is discouraged for both non‐surgical and surgical treatment of peri‐implantitis (Herrera et al. 2023), balancing benefits and disadvantages, such as swelling and rare cases of subcutaneous emphysema (Bassetti et al. 2014; Bruckmann et al. 2022; La Monaca et al. 2021; Lee et al. 2018).

Regular supportive peri‐implant care (Herrera et al. 2023) at least once a year has been demonstrated to be essential to prevent the onset of peri‐implantitis (Costa et al. 2023; Leone et al. 2024), as well as its recurrence after treatment (Monje et al. 2024). However, in vitro studies usually assess the effect of a single application of air abrasive decontamination on titanium surfaces (Moharrami et al. 2019).

Taking these aspects into consideration, the present study was developed to mimic the clinical scenario of repeated air‐polishing application overtime. The primary aim of this in vitro study was to investigate the bacterial adhesion on machined and micro‐rough titanium dental implant surfaces treated by air‐polishing with erythritol powder using single or multiple applications. The study also aimed at evaluating the effect of air‐polishing with erythritol powder on surface roughness, as well as the intrinsic antibacterial efficacy of erythritol powder.

## 2 Materials and Methods

This study was reported in accordance to the modified Consolidated Standards of Reporting Trials (CONSORT) guidelines for reporting in vitro studies on dental materials (Faggion 2012).

### 2.1 Implant Surface Treatment

Cylindrical commercially pure titanium grade IV dental implants, 4 mm in diameter and 13 mm in length, characterized by an external hexagon connection and a hybrid surface, i.e. Osseotite (OSSEOTITE) and machined (MACHINED), were used (Zimmer Biomet, Palm Beach Gardens, FL, USA).

Implant surface treatment was performed with an erythritol‐based powder presenting an average particle size of 14 μm, and 0.3% chlorhexidine (CHX) content (Airflow Plus Powder, EMS Electro Medical System, Nyon, Switzerland). An air‐polishing unit (Airflow Handy 3.0 Premium, EMS) equipped with the Perioflow nozzle (EMS) was used.

**Figure 1:**
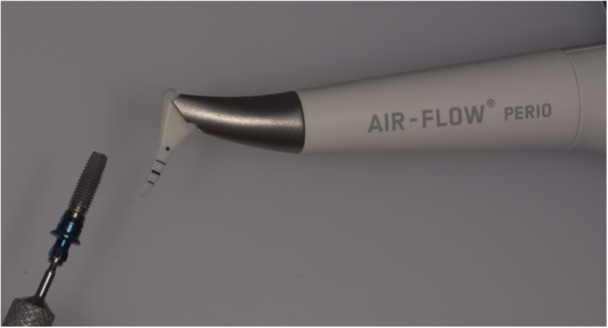
Experimental setting: Implant surface treatment by air‐polishing with erythritol powder.

The implants were divided into three groups: untreated (control), treated with one (E1) or five (E5) 1‐min cycles of continuous air‐polishing to simulate a single or repeated clinical treatments, respectively. In details, the procedure consisted in flowing the powder for 1 min keeping the nozzle parallel to the long axis of the implant, moving the handpiece along the long axis and circumferentially in order to treat the entire surface (Tastepe et al. 2017) (Figure 1). The timing had been chosen in accordance with the instructions of the manufacturer, who recommends not to exceed a treatment time of 5 s per site and an overall exposure of 1 min (E.M.S 2019; Quintero et al. 2017). After each 1‐min cycle, the implant surfaces were washed with sterile saline for 10 s, followed by 10 s of drying with compressed air. Surface characterization and microbiological analyses were conducted at the end of 1 cycle and 5 cycles in group E1 and E5, respectively.

### 2.2 Implant Surface Characterization

Surface morphological and elemental composition analyses were performed prior to microbiological tests on five implants per each test group (i.e., E1 and E5) and three for control coming from the same batch, using a FEI ESEM Quanta 200 (FEI Company, Hillsboro, OR, USA) microscope equipped with an energy dispersive X‐ray spectroscopy (EDX) detector (EDAX Element‐C2B, EDAX Inc, Mahwah, NJ, USA).

Surface topography was investigated using a stylus profilometer (Form Talysurf i‐Series, Taylor Hobson Ltd, Leicester, UK) with a radius tip of 2 μm. For all implants, on both the MACHINED and OSSEOTITE surface, three areas of 2.1 mm × 0.8 mm were scanned and four profiles of 1.8 mm per area were acquired. Ra (average roughness) and Rz (mean roughness depth) parameters were selected to describe the surface topography (ISO 4287 and ISO 25178). The profile data were filtered applying a Gaussian filter with a sampling length equal to 0.25 mm to eliminate waviness and form components. Data evaluation was performed with Talymap Contour analysis software (Taylor‐Hobson, UK).

### 2.3 Antibacterial Activity Assay

The intrinsic antibacterial activity of erythritol powder was microbiologically investigated (Belibasakis et al. 2015; Mombelli and Décaillet 2011; Scannapieco and Mylotte 1996). Methicillin‐resistant *S. aureus* (MRSA; strain 328), *Klebsiella pneumoniae* (*K. pneumoniae*; strain AIS2007023), *Streptococcus mutans* (*S. mutans*; strain NCTC10449), and *Streptococcus sanguinis* (strain DSS‐10) were purchased from ATCC (LGC Standards; Milan, Italy). Bacteria were cultured on Brain Heart infusion (BHI; *S. mutans*, *S. sanguinis*) or Trypticase Soy (TS; *K. pneumoniae*, MRSA) broth or agar.

The antimicrobial activity of erythritol powder was evaluated by determining the minimum inhibitory concentration (MIC) using the broth micro‐dilution method, standardized by the Clinical and Laboratory Standards Institute (Patel et al. 2015). Erythritol powder was dissolved in phosphate buffered saline at the initial concentration of 110 mg/mL and twofold dilutions were then prepared. Dilutions were dispersed in 96‐well micro‐titration plates and added of 1 × 10^6^ CFU/mL of single bacterial cultures growth at 37°C for 16 h. Plates were incubated for 16 h at 37°C. Absorbance was measured at 620 nm using a microplate reader (Varioskan Multimode Microplate Reader, Thermo Scientific). For each bacterial strain, the MICs were defined as the lowest concentration of erythritol at which bacterial growth was not detected. Data were confirmed by plate count procedure. Three separate experiments were carried out, each with duplicate evaluations.

### 2.4 Biofilm Inhibition Assay

To investigate the bacterial grown on control, E1 and E5 samples, five implants per treatment and bacteria strain were utilized. Implants treated with erythritol (i.e., E1 and E5) were sterilized before culture. Single cultures of *S. aureus*, *K. pneumoniae*, *S. mutans*, and *S. sanguinis* were grown for 16 h at 37°C in broth media. Implants were placed in 12‐well culture plates and inoculated with 1 × 10^4^ CFU/mL of bacterial cultures. Plates were incubated under static conditions at 37°C for 48 h (*S. aureus* and *K. pneumoniae*) or 120 h (*S. mutans* and *S. sanguinis*), time of incubation previously reported as optimal in obtaining mature biofilms (Bernabè et al. 2021; Brunello et al. 2018). In cultures of *S. mutans* and *S. sanguinis*, every 48 h the growth medium was partially replaced with fresh one.

At the end of incubation, the media was aspirated and biofilms were gently washed in sterile 0.85% sodium chloride (NaCl). Implants were transferred to 15 mL tubes, and added of 1 mL of sterile NaCl. The samples were then vigorously shaken for about 2 min, in order to ensure the detachment from the surfaces and the dispersion of the bacteria. Samples were then properly diluted and seeded on bacterial culture plates containing the appropriate agar media. The plates were incubated at 37°C for 16 h. The bacterial colonies were finally counted. For each implant, the experiment was conducted in triplicate.

### 2.5 Statistical Analysis

The literature did not offer adequate information to calculate a sample size with mathematical methods, hence the study used a convenience sample of five implants for each group of treatment and for each bacterial strain to explore the magnitude of the measurements of interest and inform the planning of further investigations.

The quantile‐quantile plot was used to check if the data followed a normal distribution. The analysis of surface data was performed with the Kruskal Wallis test with a post‐hoc analysis using Bonferroni's correction for multiple comparisons. The analysis of microbiological data was performed with repeated‐measures analysis of variance with a post‐hoc analysis using Bonferroni's correction for multiple comparisons. A *p*‐value < 0.05 was considered statistically significant. The analyses were done using SPSS 16.0 software (SPSS Inc, Chicago, IL, USA) and R 4.4 (Foundation for Statistical Computing, Vienna, Austria).

## 3 Results

### 3.1 Implant Surface Characterization

**Figure 2:**
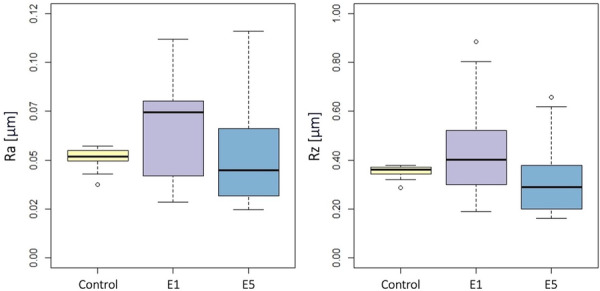
MACHINED surface: Boxplots showing Ra (left) and Rz (right) values in the three groups. Data are expressed in μm.

**Figure 3:**
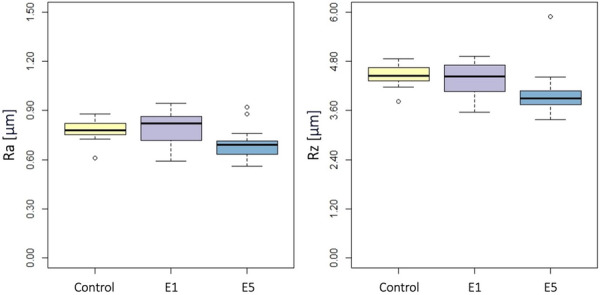
OSSEOTITE surface: Boxplots showing Ra (left) and Rz (right) values in the three groups. Data are expressed in μm.

Ra and Rz values obtained with a stylus profilometer of the MACHINED and OSSEOTITE surfaces in the three groups are presented in Table S1. On the MACHINED implant surface (Figure 2), no statistically significant differences were found between the groups in terms of Ra (*p* = 0.12) or Rz (*p* = 0.12). On the OSSEOTITE surface (Figure 3), there was a statistically significant difference between the groups in terms of Ra (*p* = 0.009) and Rz (*p* = 0.002). At the post‐hoc pairwise comparison, Ra was lower in E5 group versus E1 group (*p* = 0.03) or control group (*p* = 0.03), while there was no statistically significant difference between E1 and control groups (*p* = 0.97); Rz was lower in E5 group versus control group (*p* = 0.03), while there was no statistically significant difference between E5 and E1 (*p* = 0.07), or E1 and control groups (*p* = 0.60).

**Figure 4:**
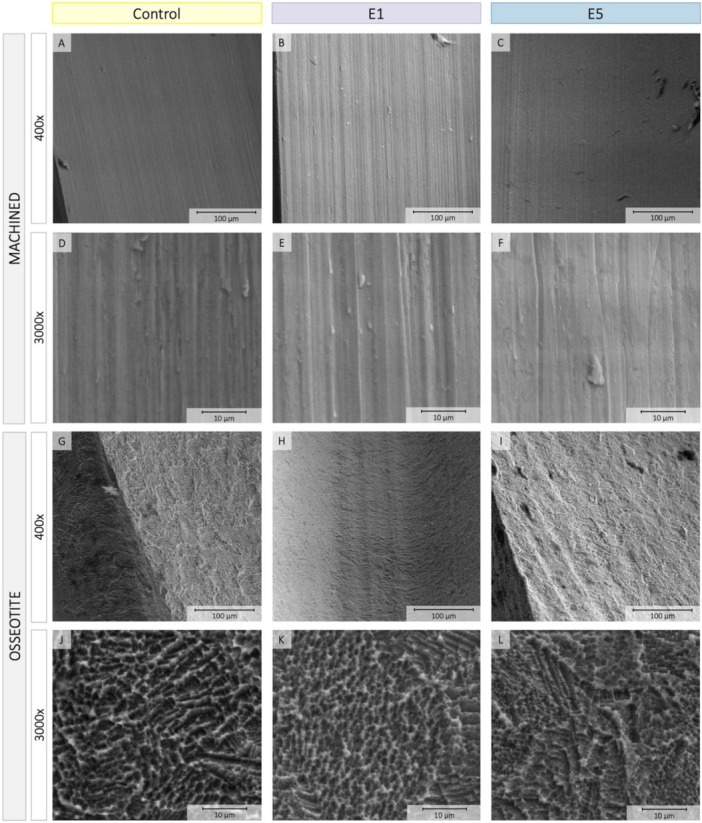
SEM images of control, E1 and E5 MACHINED (A–F) and OSSEOTITE (G–L) surfaces at two magnifications, that is 400× and 3000×.

Scanning electron microscope (SEM) analysis confirmed that both surfaces, that is MACHINED and OSSEOTITE, had not been altered by erythritol air‐polishing (Figure 4). Some residues could be observed on the implants after air‐polishing and their density appeared increased in E5 group as compared to E1 (Figure S1). EDX confirmed the presence of C and O on E1 and E5 implant surfaces, compatible with residues of erythritol.

### 3.2 Antibacterial Activity of Erythritol

The antibacterial activity of erythritol powder is summarized in Table 1. Erythritol showed an antimicrobial effect against all the tested bacterial strains. The minimal inhibitory concentrations (MICs) of erythritol ranged from 50 to 91 mg/mL. Among the four tested bacterial strains, *S. sanguinis* was the most susceptible to the antimicrobial activity of erythritol whereas *K. pneumoniae* was the most resistant. The methicillin‐resistant *S. aureus* reported a MIC value of 65 mg/mL.

**Table 1:** Minimal inhibitory concentration (MIC) of erythritol.

Bacterial strains	MIC (mg/mL)
*Staphylococcus aureus*	65
*Klebsiella pneumoniae*	91
*Streptococcus mutans*	72
*Streptococcus sanguinis*	50

### 3.3 Biofilm Reduction on Implants Treated With Erythritol

**Figure 5:**
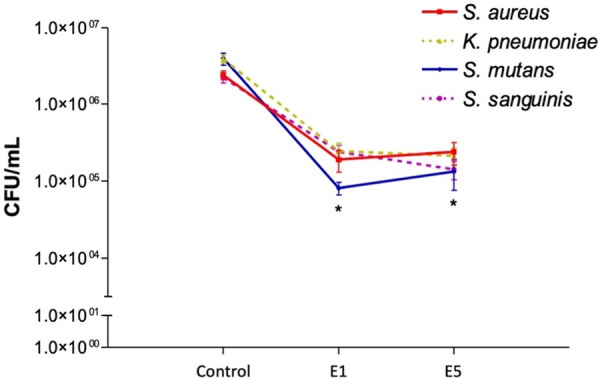
Colony counting in control, E1 and E5 group. Data are reported as mean ± standard error. * Denotes *p* < 0.05 vs. control.

To test the ability of implant surfaces treated with erythritol in reducing biofilm formation, bacteria were grown under static conditions for 48 or 120 h as described in Section Materials and Methods, 2 and bacterial viability was assessed by colony counting. As reported in Figure 5 and Table S2, E1 and E5 treatment significantly reduced the count of colonies of all tested bacterial strains as compared with bacteria cultured on implants not treated with erythritol (*p* < 0.05). No significant differences were reported between E1 and E5 samples, which were equally capable to reduce by 1.5 log the bacteria growth on the surface. Moreover, no significant differences were found in the antimicrobial effects among the four tested bacterial strains.

## 4 Discussion

Treatment with erythritol powder air‐polishing led to a substantial maintenance of the superficial features of both the examined implant surfaces, in terms of morphology, while roughness decreased on OSSEOTITE surface after multiple air‐polishing applications. Another observation was the presence of C and O on E1 and E5 implant surfaces, compatible with residues of erythritol, as detected by EDX.

The intrinsic antibacterial activity of erythritol powder as well as its biofilm inhibition effect on the tested dental implants was demonstrated. Indeed, a significative reduction of colonies count was seen between control and both E1 and E5 implants, while no significant differences were reported between E1 and E5. The inhibition in bacterial biofilm formation could be related to the intrinsic antibacterial effect of the powder rather than to the surface modification, since both E1 and E5 treated implants reported equal antibacterial effects, independently on the roughness. Indeed, pure erythritol has been shown to have an inhibitory effect on the growth of *Porphyromonas gingivalis*, *Aggregatibacter actinomycetemcomitans*, and *Actinomices viscosus* in vitro (Zhang et al. 2019).

Long‐term maintenance is critical for the preservation of dental implant health, as it prevents plaque accumulation and mitigates the risk of peri‐implant diseases (Perussolo and Donos 2024). Regular supportive peri‐implant care programmes are also crucial after the treatment of peri‐implant diseases, to reduce the risk of recurrence and ensure the stability of the results overtime (Herrera et al. 2023). To minimize surface damage during professional hygiene, mechanical debridement methods that are both gentle and effective should be applied. Indeed, aggressive debridement techniques might result in scratches or surface changes, which, in turn, could promote biofilm reaccumulation (Cha et al. 2019; Matsubara et al. 2020). Furthermore, any structural changes to the implant could lead to changes in the original design of the device resulting in the manufacturer's voiding of the warranty (Nobel Biocare 2025; ZimVie 2024).

In an in vitro study (Matsubara et al. 2020), sodium bicarbonate with its larger grain size demonstrated higher cleaning performances as compared to glycine and erythritol, whilst significantly increasing implant roughness (Sa). Similarly, other authors found that glycine and erythritol powders better preserved the integrity of the titanium surface than sodium bicarbonate (Biazussi et al. 2019; Roberto et al. 2025; Schwarz et al. 2009). In (Biazussi et al. 2019), contrary to sodium bicarbonate, air‐polishing with glycine powder did not seem to alter the surface roughness of machined titanium disks following an application time of 20 s. In (Roberto et al. 2025), damages to polished titanium surfaces were observed exclusively in the group treated with sodium bicarbonate, and not with erythritol powder. This observation aligns with our findings, with no changes detected on the machined surface after air‐polishing with erythritol powder, even after prolonged application time. Several studies have reported on residual bacterial regrowth or new bacterial colonization of implant surfaces following treatment with air‐polishing or other decontamination methods (Amate‐Fernández et al. 2021; Drago et al. 2014; Mensi et al. 2018). In the present study, we investigate the ability of different bacterial strains to adhere to surfaces treated with erythritol, specifically in the context of preventing peri‐implantitis or its recurrence. The bacterial strains selected for this study included *Streptococcus* species, *K. pneumoniae*, and *S. aureus*, all of which are commonly associated with peri‐implant infections. The antibacterial assays took into consideration aerobic bacterial species since air‐polishing is not primarily indicated in case of deep peri‐implant pockets (Herrera et al. 2023). This approach was chosen deliberately, as we did not intend to simulate the treatment of deep pockets, where anaerobic conditions typically prevail. Instead, our goal was to evaluate the efficacy of air‐polishing with erythritol in reducing bacteria colonization on implant areas that are more accessible to air‐polishing treatments, in order to prevent bacterial recolonization.

Other studies investigated the effect of air‐polishing using the same powder utilized in the present study, that is erythritol powder with a grain size of approximately 14 µm and containing 0.3% CHX (Amate‐Fernández et al. 2021; Drago et al. 2014; Mensi et al. 2018; Schmidt et al. 2017). However, unlike our study, only a single air‐polishing cycle was applied, and when specified, the duration of treatment was shorter than that used in our protocol.

Drago et al. (2014) assessed the recovery of residual biofilm after 16–18 h of incubation, finding that air‐polishing with erythritol induced a reduction of viable cells of about 50% as compared to untreated control across all tested microbial strains (i.e., *S. aureus*, *Bacteroides fragilis*, and *Candida albicans*), performing better than glycine. In a subsequent study (Drago et al. 2017), the same group confirmed the antimicrobial and antibiofilm properties of the erythritol formulation containing 0.3% CHX itself against *S. aureus*, in line with our results, as well as against *B. fragilis*, *C. albicans*, and *Pseudomonas aeruginosa*. However, in Drago et al. (2014) the lack of implant surface characterization limits the ability to discern the relative contribution of air‐polishing induced surface changes versus direct antimicrobial effects of the powder.

Mensi et al. (2018) compared the efficacy of air‐polishing with erythritol to sodium bicarbonate on grade II titanium discs. After 30 s of treatment, profilometric analysis revealed no significant changes in surface roughness among untreated controls and both test groups. Both powders prevented the adhesion and negatively affected the viability of *S. aureus* and *A. actinomycetemcomitans*, as evidenced by XTT assay and colony‐forming unit counts. Notably, erythritol demonstrated superior inhibition of bacterial re‐colonization compared to sodium bicarbonate. These findings suggest that erythritol air‐polishing is able to inhibit bacterial adhesion without altering titanium surface integrity.

Schmidt et al. (2017) evaluated the ability of various decontamination methods, including air‐polishing with erythritol and glycine powders, in preventing biofilm formation on moderately rough titanium implant necks. The authors employed not only a single‐species culture (*Streptococcus gordonii*) as in the present study, but also a mixed anaerobic culture comprising *S. gordonii*, *Actinomyces naeslundii*, *Fusobacterium nucleatum*, *Porphyromonas gingivalis*, and *Tannerella forsythia*. No significant differences in bacterial colonization were found between instrumented or control surfaces in either culture; however, SEM analysis showed that air‐polishing, particularly with erythritol, caused the least surface alterations compared to hand and ultrasonic instrumentation.

A multispecies biofilm (*P. gingivalis*, *A. actinomycetemcomitans*, *Fusobacterium nucleatum*, Actinomyces naeslundii, *Veillonella parvula,* and *S. oralis*) was utilized in another study to test the decontamination capacity and the biofilm re‐growth of two decontamination methods, that is air‐polishing with erythritol and mechanical therapy with gauze and sterile saline (Amate‐Fernández et al. 2021). Air‐polishing with erythritol inhibited biofilm regrowth to a larger extent as compared to mechanical treatment, with a significant decrease in all the investigate species except for *A. actinomycetemcomitans*, at both quantitative polymerase chain reaction (qPCR) and propidium monoazide (PMA)‐qPCR, and *S. oralis*, at PMA‐qPCR. Although *S. oralis* was also used in our study, direct comparisons remain challenging due to differing experimental conditions, particularly the use of single‐species versus multispecies biofilm models. Additionally, our study focused on colonization of previously uncontaminated surfaces, whereas Amate‐Fernández et al. (2021) examined the regrowth of residual bacteria following decontamination.

A limitation of this study is that the cleaning efficacy of erythritol was not tested. However, existing literature has already demonstrated the effectiveness of erythritol in biofilm removal from implant surfaces in vitro. In their study, Pujarern et al. (2024) reported favorable results with erythritol, showing its potential as an effective agent for biofilm removal. Its efficacy was also proved in another study comparing six powders for biofilm removal on machined (Sa=0.04 µm) and moderately rough (Sa: 1.3–1.5 µm) titanium disks (Francis et al. 2023), where air‐polishing for 20 s with erythritol powder was found to reduce biofilm autofluorescence by over 80% on both surfaces, with no significant changes to surface ultrastructure. The cleaning efficacy of erythritol was also confirmed in another work, where both erythritol and sodium bicarbonate powders were found to effectively reduce bacteria viability and number, with no significant differences between the treatments (Mensi et al. 2018). These results corroborate the findings of another study (Drago et al. 2014), with more evident biofilm reduction of *S. aureus*, *C. albicans*, and *B. fragilis* biofilms with air‐polishing with erythritol powder for an application time of 5 s as compared to air‐polishing with glycine or mechanical controls.

It is important to acknowledge that our study utilized a commercially available erythritol formulation containing 0.3% CHX, which is used, according to the manufacturer, for the preservation of the product. Given the well‐established antimicrobial properties of CHX (Alonso‐Español et al. 2025; Becker et al. 2021; Hart et al. 2024), it might have contributed to the efficacy of the treatment. Indeed, CHX has demonstrated strong antibacterial activity in vitro as a mouthwash, even at lower concentrations. A 60‐s application of 0.05% CHX combined with 0.05% cetylpyridinium chloride (CPC) or 0.1% CHX alone significantly reduced living bacteria on micro‐rough titanium surfaces (Becker et al. 2021). Similarly, in an in vitro multispecies bacterial biofilm model, a 2‐min treatment with 0.2% CHX effectively decontaminated titanium implant surfaces (Alonso‐Español et al. 2025). However, CHX did not completely eliminate biofilm biomass, underscoring its limitations in eradicating mature biofilms through rinsing alone (Alonso‐Español et al. 2025). While air‐polishing with erythritol showed antibacterial effects in the present study, to what extent CHX contributed to these effects remains unclear. Testing erythritol powder alone, without CHX, would help isolate its specific role in biofilm reduction.

Furthermore, our study did not assess whether air‐polishing with erythritol restores the biocompatibility of treated titanium surfaces. In vitro assessments, using for instance gingival fibroblasts (Schwarz et al. 2017) might provide valuable insights into how surface modifications influence cell viability and proliferation, ultimately contributing to the reestablishment of soft tissue seal around the implant (Guo et al. 2021). However, any potential cytotoxic effect might be due to the presence of CHX (Brunello et al. 2022; Weusmann et al. 2022), making it difficult to distinguish the specific impact of erythritol on biocompatibility.

Further studies would be beneficial in this context, comparing erythritol with and without CHX, offering a deeper understanding of the individual effect of erythritol alone, as well as comparing the biofilm inhibition properties of erythritol with other substances, that is glycine and sodium bicarbonate.

Our model provides the advantage of incorporating both machined portions, resembling transmucosal implant components, and micro‐rough parts, typical of implant surfaces, simulating a scenario in which the implant threads are exposed to the oral cavity. Nevertheless, the hybrid implant used in this study did not allow for separate evaluation of the microbiological outcomes in relation to surface characteristics.

Finally, given that air‐polishing is recommended for maintenance after peri‐implantitis treatments, future studies should focus on simulating professional mechanical plaque removal in patients with exposed implant surfaces following non‐regenerative surgical peri‐implantitis treatments. To this end, the effect of air‐polishing should also be evaluated on implant surfaces that have been pre‐treated with other decontamination protocols, such as NiTi rotating brushes (Brunello et al. 2025).

## 5 Conclusion

In conclusion, a reduction in colony counts was noted following air‐polishing with erythritol, regardless of the number of the applications. Based on our findings, biofilm inhibition appears to be primarily attributed to the intrinsic antibacterial properties of erythritol powder, rather than to surface modifications, as both E1 and E5 groups demonstrated a reduction in bacterial colonization, irrespective of the surface roughness. Due to its antimicrobial properties, air‐polishing with erythritol powder could represent a promising approach especially in the maintenance therapy for the prevention of peri‐implant diseases and their recurrences. However, single bacterial cultures were used in this study, and this limitation should be considered when translating the results to clinical practice. The biological environment of the oral cavity is highly complex, and the outcomes of in vitro studies may not fully represent this complexity.

## Author Contributions

**Stefano Sivolella:** conceptualization, methodology, formal analysis, investigation, resources, data curation, writing – original draft, writing – review and editing, supervision, project administration, funding acquisition. **Giulia Brunello:** methodology, formal analysis, data curation, writing – original draft, writing – review and editing. **Enrico Lotta:** formal analysis, investigation, data curation, writing – original draft, writing – review and editing. **Michele Stocchero:** formal analysis, investigation, supervision, writing – review and editing. **Roberto Meneghello:** methodology, validation, formal analysis, resources, data curation, writing – review and editing, supervision. **Paola Brun:** conceptualization, methodology, validation, formal analysis, investigation, resources, data curation, writing – review and editing, supervision.

## Ethics Statement

The authors have nothing to report.

## Conflicts of Interest

The authors declare no conflicts of interest.

## Supporting information


**Supporting Figure S1:** SEM images of control, E1 and E5 implants at 40x magnifications. Debris of erythritol powder are visible on E1 and E5 implants. **Supporting Table S1:** Summary statistics of Ra and Rz values for the MACHINED and OSSEOTITE surface in the three groups. Data are expressed in µm. **Supporting Table S2:** Colony counting in control, E1 and E5 group. Data are expressed in CFU/mL.

## Data Availability

The data underlying this article will be shared on reasonable request to the corresponding author.
