# Supplementary material for: Impact of Erythritol Air‐Polishing on Titanium Implant Surface Properties and Bacterial Colonization: An In Vitro Study
Source: Clin Exp Dent Res. 2026 Jan 14;12(1):e70289. doi: 10.1002/cre2.70289 (PMC12800741; doi:10.1002/cre2.70289)
Supplement: Supplementary file 1 — Supporting Figure S1: SEM images of control, E1 and E5 implants at 40x magnifications. Debris of erythritol powder are visible on E1 and E5 implants. Supporting Table S1: Summary statistics of Ra and Rz values for the MACHINED and OSSEOTITE surface in the three groups. Data are expressed in µm. Supporting Table S2: Colony counting in control, E1 and E5 group. Data are expressed in CFU/mL. [file CRE2-12-e70289-s001.docx]

**SUPPLEMENTARY MATERIAL**

***Suppl. Table 1.*** *Summary statistics of Ra and Rz values for the MACHINED and OSSEOTITE surface in the three groups. Data are expressed in µm.*

| **Surface** | **Group** | **Ra** | | **Rz** | |
| --- | --- | --- | --- | --- | --- |
|  |  | **Median (IQR)** | **Mean (SD)** | **Median (IQR)** | **Mean (SD)** |
| MACHINED | Control | 0.05 (0.05-0.05) | 0.05 (0.01) | 0.36 (0.34-0.37) | 0.35 (0.02) |
|  | E1 | 0.07 (0.04-0.08) | 0.06 (0.03) | 0.40 (0.30-0.52) | 0.45 (0.22) |
|  | E5 | 0.04 (0.0-0.06) | 0.05 (0.03) | 0.29 (0.20-0.38) | 0.33 (0.16) |
| OSSEOTITE | Control | 0.78 (0.75-0.82) | 0.78 (0.07) | 4.45 (4.33-4.64) | 4.45 (0.27) |
|  | E1 | 0.82 (0.72-0.86) | 0.78 (0.10) | 4.43 (4.07-4.70) | 4.36 (0.44) |
|  | E5 | 0.69 (0.63-0.71) | 0.69 (0.10) | 3.90 (3.74-4.08) | 3.99 (0.60) |

***Suppl. Figure 1.*** *SEM images of control, E1 and E5 implants at 40x magnifications. Debris of erythritol powder are visible on E1 and E5 implants.*


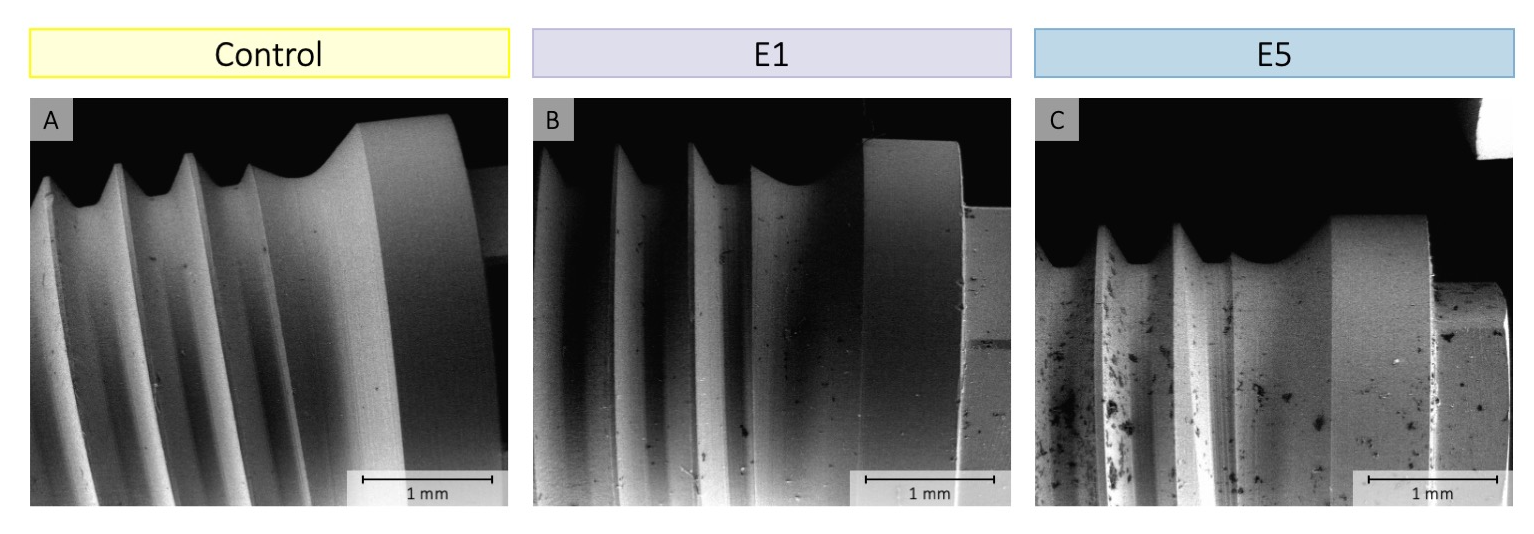


***Suppl. Table 2.*** *Colony counting in control, E1 and E5 group. Data are expressed in CFU/mL.*

| **Bacterium** | **Group** | **Median** | **Mean** | **SD** |
| --- | --- | --- | --- | --- |
| *S. aureus* | Control | 2160000 | 2380000 | 312780 |
|  | E1 | 107500 | 190223 | 60002 |
|  | E5 | 73000 | 239447 | 77228 |
| *K. pneumoniae* | Control | 3586000 | 3856000 | 458271 |
|  | E1 | 164000 | 244320 | 67040 |
|  | E5 | 154000 | 212913 | 54373 |
| *S. mutans* | Control | 2800000 | 3946000 | 760062 |
|  | E1 | 108000 | 81155 | 14828 |
|  | E5 | 62000 | 132854 | 56951 |
| *S. sanguinis* | Control | 1605000 | 2207000 | 334067 |
|  | E1 | 305000 | 239957 | 49703 |
|  | E5 | 83000 | 141551 | 37970 |
